# Editorial: Spatial-temporal metrics to assess collective behavior in team sports

**DOI:** 10.3389/fpsyg.2025.1743592

**Published:** 2025-12-08

**Authors:** Antonio García-de-Alcaraz, Yixiong Cui, Bingnan Gong, Tianbiao Liu

**Affiliations:** 1 University of Almeria, Almería, Spain; 2 Beijing Sport University, Beijing, China; 3 Capital University of Physical Education and Sports, Beijing, China; 4 Beijing Normal University, Beijing, China

**Keywords:** tactical behavior, decision-making, performance analysis, sport dynamics, complexity

The analysis of collective behavior in team sports has advanced through the integration of spatiotemporal, technical, cognitive, and computational approaches that aim to describe how teams organize, interact, and adapt under dynamic conditions. The articles compiled in this Research Topic provide a comprehensive overview of these perspectives, spanning different sports and levels of expertise. By combining observational, modeling, and experimental methods, this collection highlights the potential of spatial-temporal metrics to bridge theoretical understanding and applied practice, strengthening the connection between scientific evidence and performance optimization.

Studies focusing on technical–tactical variables aimed to identify which performance indicators most strongly predict success across different team sports. These works quantified the contribution of efficiency in specific phases of play, such as attack and counterattack in beach volleyball (da Costa et al.), or the structure and flow of ball possessions in elite football (Maneiro et al.). Similarly, the investigation of tactical dynamics in Guardiola's teams (Pueyo et al.) revealed how match context influences collective organization. These studies collectively emphasize how match outcomes are determined by the quality and coordination of technical execution within specific tactical phases, reinforcing the value of data-driven indicators for assessing team effectiveness.

A second group of contributions explored the spatial–temporal coordination that underpins collective performance. Using positional and tracking-based analyses, these studies examined how inter-player distances, team dispersion, and synchronization evolve across game phases. Experimental research in 3 × 3 basketball (Ichikawa et al.) demonstrated that explicit tactical cues enhance spacing and offensive coordination, while comparative analyses in youth football (Liang et al.) showed that spatial pressure and interpersonal distance differentiate tactical maturity between cultures. Together, these works confirm that spatial-temporal organization—how teams occupy, share, and adjust space—serves as a core determinant of collective adaptability and effectiveness.

Several studies advanced the use of computational and data-driven methods to model and predict collective behavior. The review by Sotudeh synthesized the methodological foundations for automated formation detection in football, emphasizing the transition from static to dynamic representations of team structure. Complementarily, machine learning applications in the FIFA Women's World Cup 2023 (Iván-Baragaño et al.) and Qatar 2022 (Song et al.) predicted shooting and match outcomes using a range of spatial-temporal and technical indicators. These works share a common goal: to transform large-scale tracking and event data into interpretable models that support tactical decision-making and performance prediction in real competition.

Another dimension addressed concerns the perceptual and cognitive mechanisms underlying team coordination and skilled performance. Habekost et al. proposed a comprehensive model describing the perception–action cycle in elite soccer, integrating attention, anticipation, and feedback processes. Similarly, Piras empirically examined gaze behavior during basketball three-point shots, showing how experts modulate fixation and saccadic control to optimize information use under time constraints. These studies converge in recognizing that perceptual attunement and cognitive control are critical components of collective dynamics, linking visual information processing with decision-making efficiency.

Finally, other contributions analyzed how contextual and structural variables condition collective organization and performance. Lee and Kim examined the impact of functional classification and team composition in wheelchair basketball, revealing the importance of balanced line-ups for optimizing play efficiency. Comparative analyses of match location and cultural background (Pueyo et al.; Liang et al.) further demonstrated that environmental and situational contexts shape how teams coordinate space and adapt strategies. These findings highlight that collective behavior is not only the result of internal team dynamics but also a reflection of structural, environmental, and cultural influences that frame performance in real settings.

Overall, the contributions within this Research Topic illustrate the growing maturity and diversification of research on collective behavior in team sports. Across different disciplines, these studies demonstrate that spatial-temporal metrics, technical–tactical indicators, perceptual–cognitive mechanisms, and computational modeling converge toward a shared goal: explaining how coordination, adaptation, and decision-making emerge from complex interactions among players and their environments. However, this body of work also exposes persistent limitations. Despite methodological advances, there is still a need for standardized metrics and cross-sport validation frameworks that allow comparison and replication across contexts. The ecological validity of many experimental or data-driven designs remains challenging, as does the integration of physical, tactical, and cognitive dimensions into unified models. Moreover, the translation of large-scale tracking data into accessible tools for coaches and practitioners continues to be limited by technological and interpretative barriers. Addressing these gaps is essential to consolidate a coherent, evidence-based understanding of collective behavior.

Future research should move toward the integration of multimodal data sources—including positional, inertial, physiological, and perceptual information—to capture the full spectrum of interactions that define team performance. Combining these datasets with advanced analytical techniques such as machine learning, network theory, and non-linear modeling will enable the development of predictive frameworks that reflect the dynamic and adaptive nature of sport. At the applied level, spatial-temporal metrics hold enormous potential to transform training design and tactical preparation, providing coaches with objective indicators of team cohesion, spacing efficiency, and decision-making speed. Embedding these analyses within the daily training process could enhance players' situational awareness, collective synchronization, and perceptual–cognitive expertise, bridging the gap between scientific knowledge and professional practice.

